# Efficient Immortalization of Primary Nasopharyngeal Epithelial Cells for EBV Infection Study

**DOI:** 10.1371/journal.pone.0078395

**Published:** 2013-10-22

**Authors:** Yim Ling Yip, Pei Shin Pang, Wen Deng, Chi Man Tsang, Musheng Zeng, Pok Man Hau, Cornelia Man, Yuesheng Jin, Anthony Po Wing Yuen, Sai Wah Tsao

**Affiliations:** 1 Cancer Biology Laboratory, Department of Anatomy, Li Ka Shing Faculty of Medicine, the University of Hong Kong, Pokfulam, Hong Kong SAR, China; 2 School of Nursing, Li Ka Shing Faculty of Medicine, the University of Hong Kong, Pokfulam, Hong Kong SAR, China; 3 State Key Laboratory of Oncology in South China, Sun Yat-sen University Cancer Center, Guangzhou, China; 4 Department of Applied Biology and Chemical Technology, the Hong Kong Polytechnic University, Hong Kong SAR, China; 5 Department of Clinical Genetics, Lund University, Lund, Sweden; 6 Hong Kong Head and Neck Ear Nose Throat Surgery Centre, Hong Kong SAR, China; The University of North Carolina at Chapel Hill, United States of America

## Abstract

Nasopharyngeal carcinoma (NPC) is common among southern Chinese including the ethnic Cantonese population living in Hong Kong. Epstein-Barr virus (EBV) infection is detected in all undifferentiated type of NPC in this endemic region. Establishment of stable and latent EBV infection in premalignant nasopharyngeal epithelial cells is an early event in NPC development and may contribute to its pathogenesis. Immortalized primary nasopharyngeal epithelial cells represent an important tool for investigation of EBV infection and its tumorigenic potential in this special type of epithelial cells. However, the limited availability and small sizes of nasopharyngeal biopsies have seriously restricted the establishment of primary nasopharyngeal epithelial cells for immortalization. A reliable and effective method to immortalize primary nasopharyngeal epithelial cells will provide unrestricted materials for EBV infection studies. An earlier study has reported that *Bmi-1* expression could immortalize primary nasopharyngeal epithelial cells. However, its efficiency and actions in immortalization have not been fully characterized. Our studies showed that *Bmi-1* expression alone has limited ability to immortalize primary nasopharyngeal epithelial cells and additional events are often required for its immortalization action. We have identified some of the key events associated with the immortalization of primary nasopharyngeal epithelial cells. Efficient immortalization of nasopharyngeal epithelial cells could be reproducibly and efficiently achieved by the combined actions of *Bmi-1* expression, activation of telomerase and silencing of *p16* gene. Activation of MAPK signaling and gene expression downstream of *Bmi-1* were detected in the immortalized nasopharyngeal epithelial cells and may play a role in immortalization. Furthermore, these newly immortalized nasopharyngeal epithelial cells are susceptible to EBV infection and supported a type II latent EBV infection program characteristic of EBV-infected nasopharyngeal carcinoma. The establishment of an efficient method to immortalize primary nasopharyngeal epithelial cells will facilitate the investigation into the role of EBV infection in pathogenesis of nasopharyngeal carcinoma.

## Introduction

Nasopharyngeal carcinoma (NPC) is a common cancer among southern Chinese. It is closely associated with Epstein-Barr virus (EBV) infection [1]. Immortalized nasopharyngeal epithelial (NPE) cells generated from high risk population (Cantonese) will be valuable tools to study EBV infection and its role in the NPC pathogenesis. Access to non-malignant NPE tissues is extremely limited and surgically biopsied nasopharyngeal tissues are small in size; hence presenting tremendous challenges to establish immortalized NPE cells for EBV infection study. Establishment of an efficient and reliable method to immortalize primary NPE cells will greatly facilitate research study in NPC. Viral oncogenes, notably SV40T and combined action of E6 and E7 from high risk HPV (type 16 and 18), have been commonly used in cell immortalization. In combination with telomerase, high efficiency of immortalization could be achieved. The viral oncogenes could effectively inactivate G1/S cell cycle checkpoint through inactivation of p53 and Rb proteins, releasing cells to progress into cell cycle. The expression of human telomerase reverse transcriptase (hTert) further compensates the continuous erosion of telomere in dividing cells to prevent onset of cellular senescence; and in combination with either SV40T or HPV16E6/E7 could effectively immortalize many types of human cells. Our laboratory has previously achieved immortalization of NPE cells using either *SV40T* or *HPV16 E6/E7* alone [2]. The process of immortalization was long and the success rate was low. Furthermore, neither SV40 nor HPV has been implicated in the pathogenesis of NPC. The presence of these viral oncogenes may interfere with the actions of EBV encoded products and limit their applications for study of EBV infection in NPC pathogenesis. Immortalization of NPE cells has been achieved by expression of hTert alone but occurred at a very low efficiency [3]. A more efficient and reliable method to immortalize NPE cells remains to be sought.

*Bmi-1* represents a good choice for immortalization of primary NPE cells. It is commonly overexpressed in NPC and could be detected in 38.7% of NPC biopsies [4].. Hence, NPE cells immortalized by Bmi-1 will be more representative cell model for EBV infection study. While the immortalization ability of *Bmi-1* in primary NPE cells has been demonstrated in an earlier study [4], detailed examination of events associated with the immortalization of NPE cells by *Bmi-1* have not been characterized. In this study, we have examined in details the efficiency of *Bmi-1* to immortalize primary NPE cells and have characterized some of the crucial events underlying its immortalization action. An efficient method to immortalize primary NPE cells by combined actions of *Bmi-1, hTert* and silencing of *p16* was also established. Furthermore, primary NPE cells immortalized by this protocol were shown to be susceptible to EBV infection which will facilitate their applications in the study of EBV infection in NPC pathogenesis. 

The *Bmi-1* belongs to the polycomb group family [5] which remodels chromatin protein and deregulates genes commonly involved in carcinogenesis [6,7]. In earlier reports, Bmi-1 was reported to suppress the expression of p16 and p14 at the *INK4A* locus to overcome cellular senescence [8]. Downregulation of *Bmi-1* suppressed proliferation of lymphoma cells. Telomerase activity was detected in cells overexpressing *Bmi-1* [9]. The abilities to inhibit p16 and activate telomerase are considered to be the two major properties of *Bmi-1* to immortalize human epithelial cells. However, the immortalization actions of Bmi-1 appear to differ among human epithelial cells from different tissue origins. While weak telomerase activity was reported in human mammary epithelial cells upon introduction of *Bmi-1*, telomerase activity was not commonly detected in dermal keratinocytes and small airway epithelial cells [10]. Suppression of p16 expression was observed when *Bmi-1* was overexpressed in human mammary epithelial cells and was attributed for the extension of *in vitro* lifespans of human mammary epithelial cells [9,10]. However, another study showed that *Bmi-1* expression in oral keratinocytes effectively extended *in vitro* lifespan without significant reduction of p16 expression [11]. Events underlying *Bmi-1* immortalization of primary NPE cells are largely undefined. Characterization of these events will facilitate the efficient immortalization of this special type of epithelial cells for NPC study. 

## Materials and Methods

### Cell culture and cell lines

Primary NPE cells (NP105, NP361, NP446 and NP550) were established by explanting small size biopsies of non-malignant nasopharyngeal tissues from patients admitted to the Queen Mary Hospital, University of Hong Kong, Hong Kong. Prior written informed consents for the use of these tissues for research investigation were obtained from patients or guardians on the behalf of the children participants. The collection and use of these specimens have been approved by the Human Research Ethic Committee of the University of Hong Kong. The biopsy of NP105 was taken from tonsil of a 4-year-old female and the tissue of NP550 was taken from the right nasopharyngeal region of a 76-year-old male with NPC and was examined to be tumor free before use for explant culture. Patients information of NP361 and NP446 biopsies have been described [12]. Details of the culture methods have been previously described [2,3]. The NPE2 Bmi-1 cell was an immortalized NPE cell line and the detail has been published [4]. Akata-EBV and a NPC cell line (C666-1) were cultured in RPMI-1640 medium (Sigma, St. Louis, MO) supplemented with 10% fetal bovine serum (GIBCO, Invitrogen, Carlsbad, CA), 100 U/ml penicillin and 100 μg/ml streptomycin. All the cells were maintained in a 37 °C incubator with 5% CO_2_ in air.

### Expression of Bmi-1 and hTert in NPE cells

Infective retrovirus was prepared according to previous publications [3,4,13]. The cells after transduction with *Bmi-1* were selected with 100 ng/ml puromycin for 2 weeks. The expression of *Bmi-1* was confirmed by Western blot analysis. The human telomerase reverse transcriptase (*hTert*) gene was then transduced into the Bmi-1-expressing cells. Details for the transduction of *hTert* and detection of telomerase activity were previously described [3].

### Immunofluorescence staining for cytokeratin

Immunofluorescent staining of acidic cytokeratin (clone AE1; Zymed Laboratories, Inc.) and basic cytokeratin (clone AE3; Zymed Laboratories, Inc.) was performed as previously described [12].

### Cytogenetic analysis of the immortalized NPE cells

Spectral karyotyping was performed as previously described [14].

### Flow cytometric analysis

Cells were fixed in ice-cooled 70% ethanol. After replacing with phosphate buffered saline, the suspension was incubated with 10 μg propidium iodide and 10 μg RNase A at 37°C for 20 minutes. The flow cytometry data was acquired using FACS CantoII flow cytometer (BD Biosciences, San Jose, CA, USA) and the results were analyzed by ModFit LT2.0 software (Coulter Electronics). EBV infection rate in the immortalized NPE cells was determined based on the GFP expression after 2 days infection. Cell suspension was analyzed by LSR Fortessa Analyzer (BD Biosciences).

### Western blotting analysis

Details procedures of Western blot analysis have been published previously [15]. The primary antibodies used were: actin (I-19; 1:1000; Santa Cruz Biotechnology, Santa Cruz, CA), anti-vimentin (VIM 3B4; 1:1000; Boehringer Mannheim, Germany), Bmi-1 (F6; 1:200; Millipore), Cytokeratin 5/6/18 (LP34; 1:1000; Novocastra Laboratories, Newcastle, UK), Cytokeratin 8 (4.1.18; 1:1000; Chemicon, Temecula, CA), Cytokeratin 13 (Ks 13.1; 1:1000; Chemicon), Cytokeratin 19 (b170; 1:1000; Novocastra Laboratories), EGFR (1005; 1:500; Santa Cruz Biotechnology), p16 INK4A (1:500; Cell Signaling Technology, Danvers, MA), p21 (F-5; 1:500; Santa Cruz Biotechnology), p53 (DO-7; 1:2000; Dako, Demmark), phospho-p44/42 MAPK (Erk1/2) (Thr202/Tyr204) (1:500; Cell Signaling Technology) and phospho-MEK1/2 (Ser217/221) (1:500; Cell Signaling Technology). 

### RNA extraction and RT-PCR analysis

Total RNA was extracted from the culturing cells at their exponential growth phase, reversely transcribed to cDNA and subjected to PCR analysis as previously described [13]. The primers sequences for the genes in Bmi-1 driven pathways and for the EBV-encoded transcripts have been published [16,17]. 

### Bio-Plex phosphoprotein assay

Quantitative detection of phosphorylated protein of Akt (Ser^473^), JNK (Thr^183^/Tyr^185^), MEK1 (Ser^217^/Ser^221^), NFκB p65 (Ser^536^), p38 MAPK (Thr^180^/Tyr^182^), Stat3 (Tyr^705^) and Stat6 (Tyr^641^) in cell lysate was performed using a bead-based Bio-Plex phosphoprotein assay kit (Bio-Rad Laboratories, Hercules, CA). Details procedures have been described [12].

### Knocking down p16 in NPE cells

Lentiviral vector for silencing of p16 (pRRL.SIN-18shp16) was kindly provided by Prof. Judith Campisi at the University of California, USA. Lentivirus was prepared by cotransfecting p16 silencing vector with a lentivirus packaging vector mix (Invitrogen) into the 293T packaging cell line. The lentivirus-containing supernatant and hexadimethrine bromide (4 μg/ml) (Sigma) were added to the NPE cells. The silencing of *p16* was confirmed by Western blot analysis.

### EBV infection of NPE cells

EBV infection of immortalized NPE cells was achieved by either coculturing of NPE cells with lytically-induced EBV-infected Akata cells or incubating the NPE cells with cell-free EBV supernatant. The experimental procedures for the coculture with Akata cells have been described previously [18]. For the cell-free EBV infection, supernatant from the lytically-induced Akata cells was harvested. Epithelial cells were incubated with the supernatant with centrifugation.

## Results

### Efficiency of Bmi-1 to immortalize primary NPE cells

We have examined the immortalization property of *Bmi-1* in primary cultures of NPE cells. The impact of *Bmi-1* on the cellular and molecular properties of the immortalized NPE cells and the susceptibility of *Bmi-1*-immortalized cells to EBV infection were characterized. Primary NPE cell cultures used in this immortalization study were established from non-malignant nasopharyngeal biopsies obtained from NPC patients and tonsillectomies (Table S1 in File S1). The course and events occurred during immortalization of four primary cultures of non-malignant nasopharyngeal cultures were carefully examined. Two of these primary cultures, NP361 and NP550, were established from non-malignant nasopharyngeal epithelial biopsies from NPC patients. The remaining two primary cultures, NP105 and NP446, were established from tonsillectomized tissues from non-NPC patients. All these nasopharyngeal biopsies were confirmed by histopathological examination to be tumor free. Spectral karyotyping analysis in primary nasopharyngeal epithelial cells from these biospies revealed normal and diploid karyotype (46 chromosomes) with no cytogenetic aberrations (data not shown).

Similar to primary cultures of other human epithelial cells, these non-malignant NPE cells all underwent cellular senescence as evidenced by their enlarged cell morphology and expression of senescence-associated beta galactosidase (data not shown). The number of passages that could be achieved before onset of cellular senescence varied among the four primary NPE cultures (Table S1 in File S1). The two primary NPE cultures established from non-malignant nasopharyngeal biopsies obtained from NPC patients (NP361 and NP550) could be propagated for a longer passage (PD 20 and PD 24 respectively) before onset of cellular senescence while the primary NPE cells derived from non-NPC patients (NP105 and NP446) underwent cellular senescence at a much shorter passage in culture (PD 6 and PD 10 respectively) (Table S1 in File S1). This may indicate the presence of occult genetic alterations in tumor adjacent nasopharyngeal epithelial tissues supporting a longer *in vitro* lifespan. A larger sample size will be warranted for this observation to be conclusive. 

**Figure 1:**
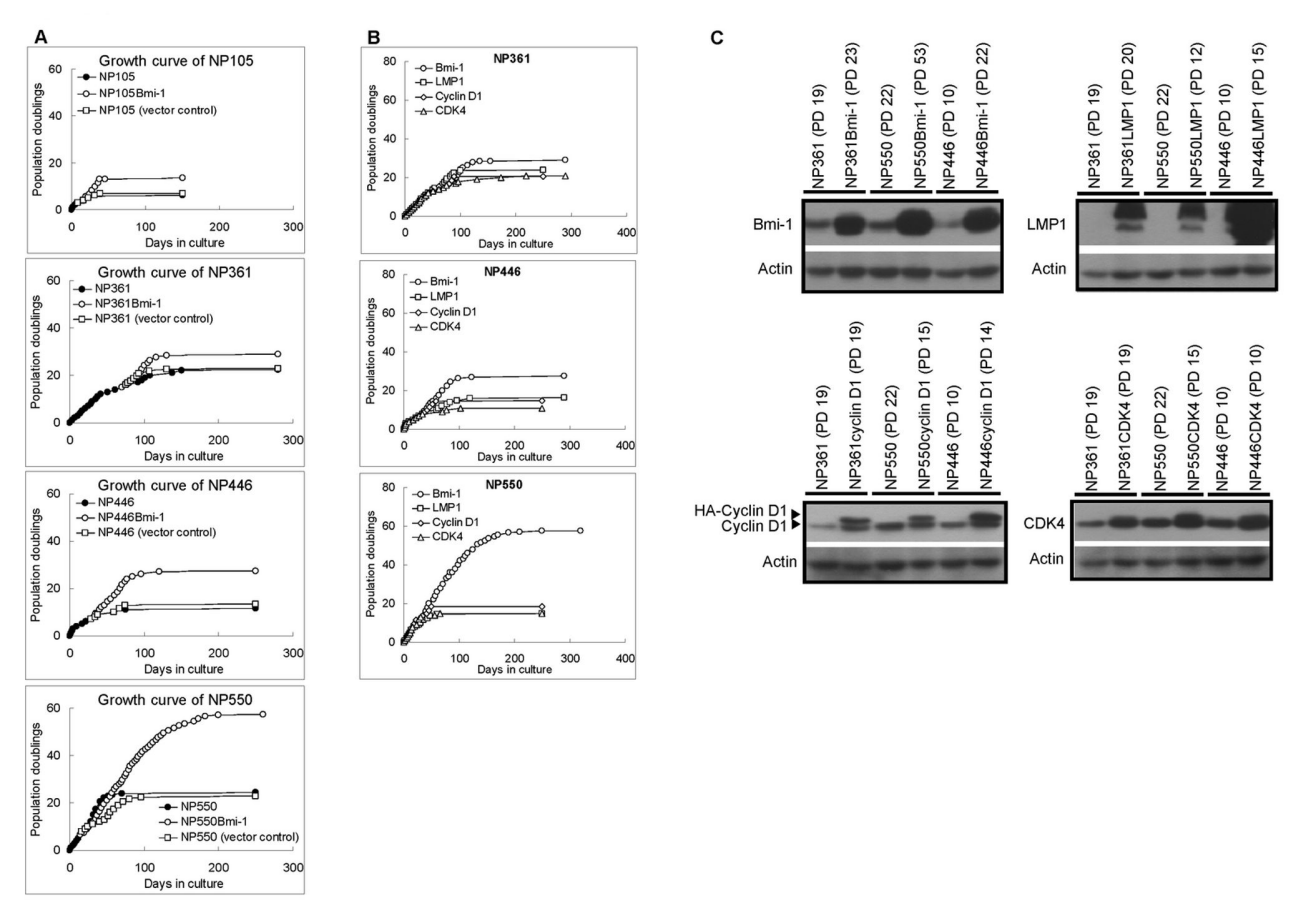
Growth history of primary nasopharyngeal epithelial cells. (A) Immortalization history of primary nasopharyngeal epithelial cells by Bmi-1. NP105, NP361, NP446 and NP550 stopped proliferation at population doublings 6 to 24. The cells were subjected to immortalization by transduction with Bmi-1. The introduction of Bmi-1 could extend the life span of the cells. (B) Expression of genetic elements in primary nasopharyngeal epithelial cells. Genetic elements, Bmi-1, LMP1, cyclin D1 and p16-insensitive form of CDK4, were expressed in primary nasopharyngeal epithelial cells. Among them, Bmi-1 was the most efficient in extension of lifespan of cells. (C) Ectopic expression of Bmi-1, LMP1, cyclin D1 and CDK4 in the primary nasopharyngeal epithelial cells. The cells could maintain the expression of the exogenous genes until the late stage as confirmed by Western blot.

*Bmi-1* was ectopically expressed in these four non-malignant NPE cell cultures and examined for its ability to immortalize them (Figure 1A). In all the four primary NPE cultures, expression of *Bmi-1* successfully delayed their onset of cellular senescence and markedly extended their *in vitro* lifespans. However, none of these *Bmi-1*-expressing NPE cell cultures become immortalized suggesting that additional events are required in the immortalization of NPE cells by *Bmi-1*. The numbers of population doubling (PD) achieved in various primary cultures after expression of Bmi-1 were 6, 29, 27 and 57 respectively for NP105, NP361, NP446 and NP550. 

We have also compared the ability of *Bmi-1* to extend *in vitro* lifespan of primary NPE cells with other genetic elements including *LMP1*, *cyclin D1* and *CDK4* (Figure 1B and Table S2 in File S1). Their ectopic expressions in primary nasopharyngeal epithelial cells were confirmed by Western blotting (Figure 1C). The *LMP1* is an EBV-encoded oncogene which has previously been reported by our group to have ability to immortalize human NPE cells in combination with telomerase [12]. Overexpression of *cyclin D1* or *CDK4*, in combination with telomerase and other genetic alterations such as *EGFR*, has been reported by others to immortalize oral keratinocytes [19]. The *CDK4* used in this study is a mutant form (CDK4^R24C^), harboring a mutation of arginine to cysteine at codon 24, rendering the cells to be insensitive to growth inhibitory action of p16. Compared to these genetic elements, *Bmi-1* was shown to be the most efficient in extending the *in vitro* lifespan of primary NPE cell cultures (Figure 1B and Table S2 in File S1). 

### Immortalization of primary NPE cells by combined actions of Bmi-1 and hTert

**Figure 2:**
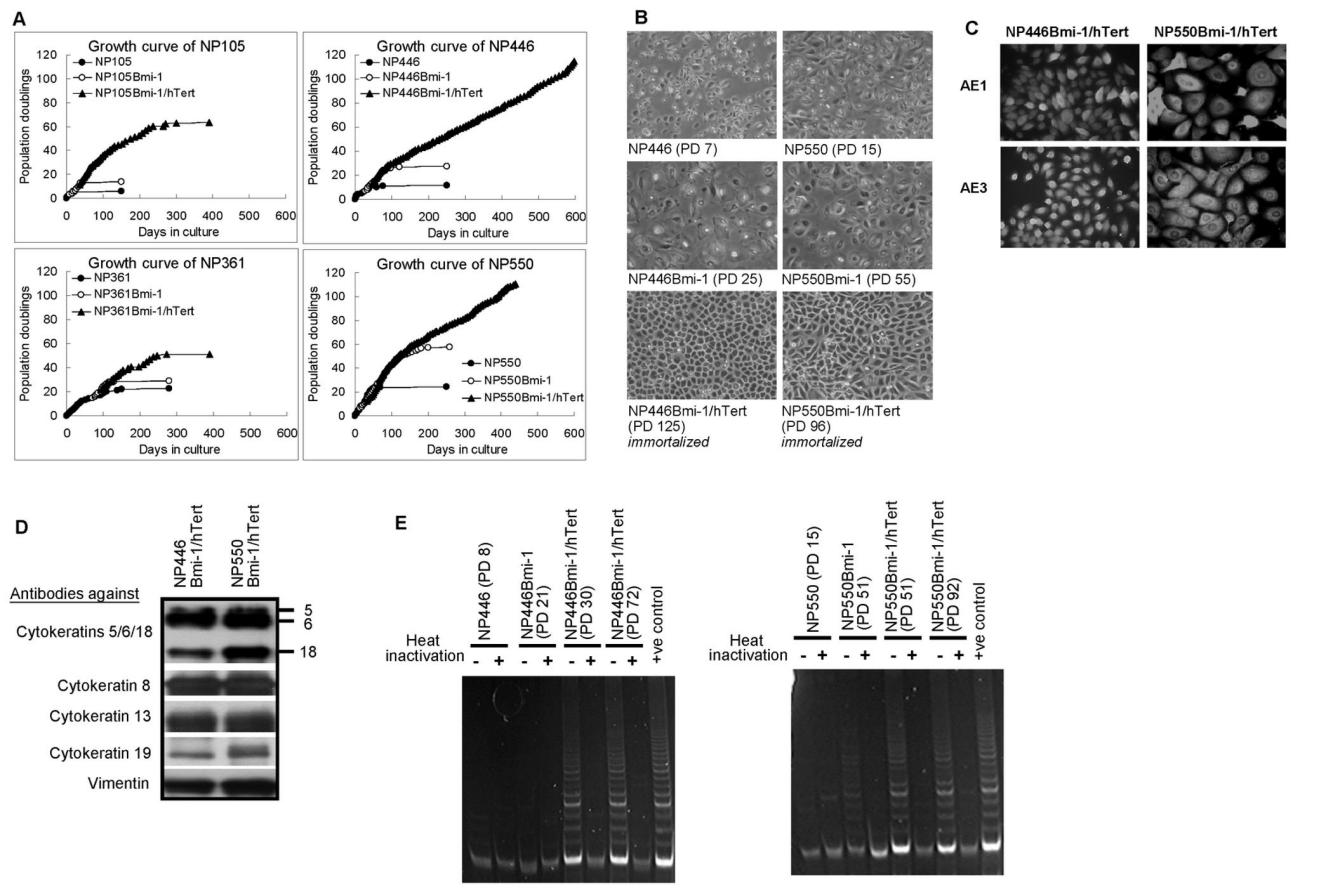
Properties of *Bmi-1/hTert*-immortalized cells. (A) Immortalization of nasopharyngeal epithelial cells by expression of Bmi-1 and activation of telomerase. Immortalization could be observed in NP446 and NP550 cell lines by expression of Bmi-1 together with hTert. (B) Microscopic images of normal human nasopharyngeal epithelial cells and immortalized nasopharyngeal epithelial cells (240x). Normal cells of NP446 and NP550, at PD 7 and 15 respectively. They were still proliferative. The normal cells after introduction of Bmi-1 alone still became enlarged and stopped proliferating at later passage. The introduction of Bmi-1 together with hTert to the normal cells extended their life span in culture and the cells were immortalized. (C-D) The epithelial origins of the cells are confirmed by expression of cytokeratin. (C) Immunocytochemistry staining of cytokeratin. (D) Western blot confirmed expression of cytokeratin. (E) Telomerase activity by TRAP assay. Successful induction of telomerase activity in immortalized cells after introduction of hTert. Bmi-1 alone could also induce the telomerase activity in NP550 cell line. However, a relatively low telomerase activity could be observed in the cells after the introduction of Bmi-1 alone or the cells from primary culture.

We then sought for the additional crucial elements required for immortalization of primary NPE cells by *Bmi-1*. Telomerase activation is critical for cell immortalization. The ability of Bmi-1 in combination with *hTert* (catalytic unit of telomerase) to immortalize primary cultures of NPE cells was then examined. Our previous studies have showed that immortalization of NPE cells by telomerase alone is a rare event and additional genetic alterations are involved [3,12]. In this study, expression of telomerase alone also failed to immortalize these four primary NPE cell cultures despite the successful activation of telomerase by *hTert* expression (Fig. S1). In combination, *Bmi-1* and *hTert* successfully immortalized two (NP446 and NP550) out of the four primary cultures of NPE cells examined (Figure 2A). The lifespan of the remaining two primary NPE cell cultures (NP105 and NP361) was also markedly extended by the combined action of *Bmi-1* and *hTert* (Figure 2A). 

### Characterization of NPE cells immortalized by Bmi-1 and hTert

The properties of these two newly immortalized NPE cells are described here. The *Bmi-1/hTert*-immortalized NPE cell lines (NP446 and NP550) are non-tumorigenic when injected subcutaneously to BALB/cAnN-nu (Nude) mice at 10^6^ to 10^7^ cells per mouse and observed for 3 to 6 months period. Under phase contrast microscope, the *Bmi-1/hTert*-immortalized cells appeared smaller in size compared to their parental cells (Figure 2B). The epithelial nature of the immortalized NP446 and NP550 cells was confirmed by the presence of keratin revealed by immunocytochemical staining using keratin-specific antibodies (AE1 and AE3) (Figure 2C). Western blotting analysis of keratin profiles of *Bmi-1/hTert*-immortalized NPE cells revealed the presence of keratins 5, 6, 8, 13, 18 and 19 (Figure 2D) which are the common keratin profile expressed in primary NPE cells [2]. 

### Ability of Bmi-1 to induce telomerase activity in human NPE cells

Previous study has reported that *Bmi-1* could induce telomerase activity in human mammary epithelial cells to facilitate cell immortalization [9]. We then examined if *Bmi-1* could activate telomerase activity in our NPE cells by the sensitive TRAP assay (Figure 2E). Telomerase activity was not detectable in NP446 cells after expression of *Bmi-1* (NP446Bmi-1). A low level of telomerase activity was however detected in NP550 cells after expression of *Bmi-1* (NP550Bmi-1). However, the low level of telomerase detected in NP550Bmi-1 cells was not sufficient to achieve immortalization as NP550Bmi-1 continued to undergo cellular senescence. Telomerase activity was also not detected in NP105 and NP361 cells expressing Bmi-1 alone (data not shown). In contrast, robust telomerase activity was detected in the *Bmi-1/hTert*-immortalized NPE cells (NP446Bmi-1/hTert and NP550Bmi-1/hTert cells). We can conclude that Bmi-1 expression is not efficient to activate telomerase in primary NPE cells to achieve immortalization.

### Karyotypic analysis of the Bmi-1/hTert-immortalized NPE cells

**Figure 3:**
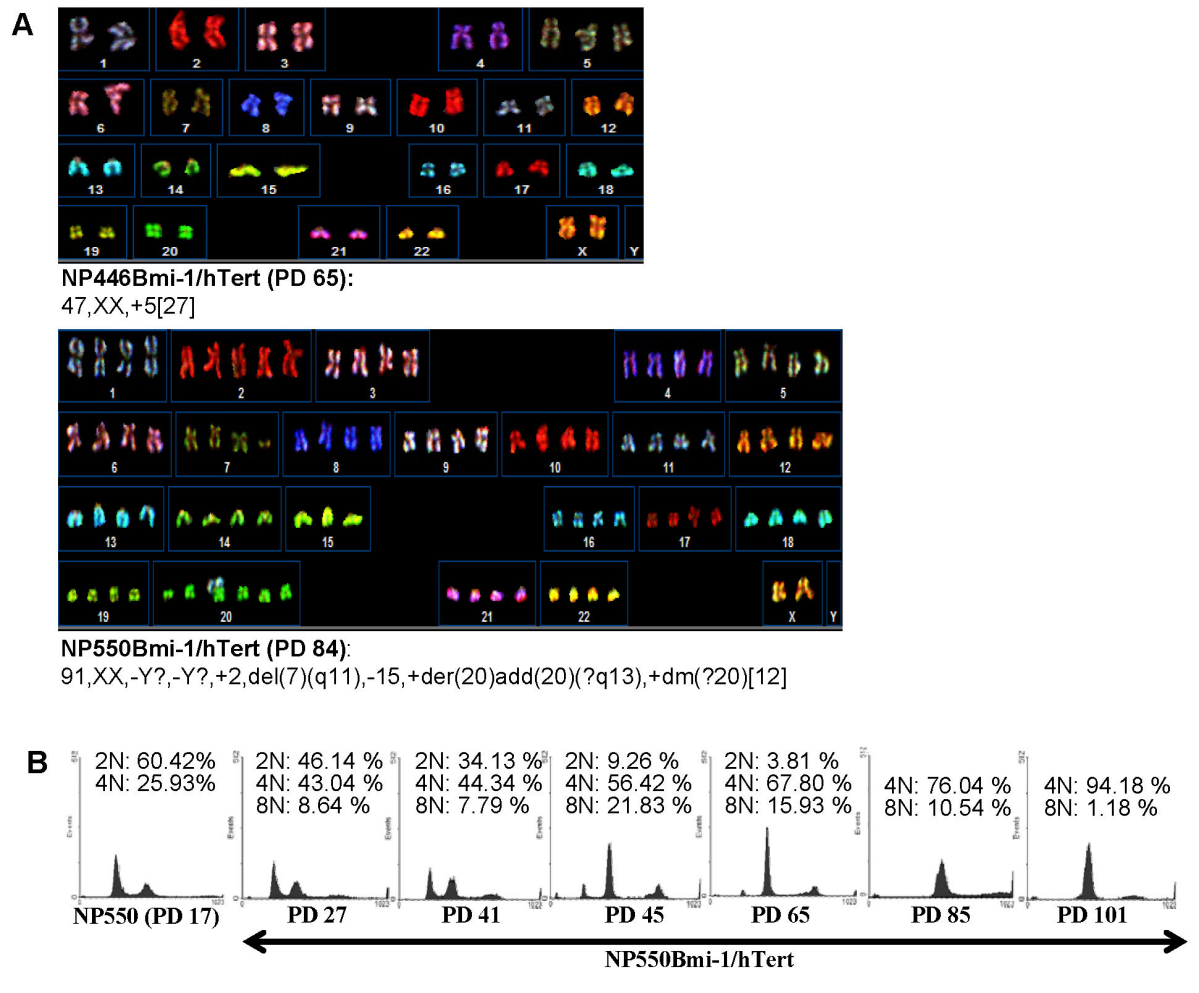
Karyotypic analysis of the immortalized NPE cells. (A) Spectral karyotyping of the major clones in the Bmi-1 immortalized cells. Majority of clones in NP446Bmi-1/hTert are quite normal and only contained one extra chromosome 5. NP550Bmi-1/hTert are nearly-tetraploid. (B) Flow cytometry analysis of normal NP550 cells and NP550Bmi-1/hTert immortalized cells at different stage of immortalization. Cell cycle distribution of the nasopharyngeal epithelial cells derived from primary culture and different passages of the NP550Bmi-1/hTert immortalized cells revealed by flow cytometry. A switch to tetraploid could be detected after expression of Bmi-1 in NP550. In NP550Bmi-1/hTert, a switch to tetraploid could be detected as early as at PD 27.

Detailed examination by spectral karyotyping analysis reveals the presence of some chromosomal abnormalities in the two *Bmi-1/hTert*-immortalized NPE cells. The abnormalities are mostly numerical in nature. The karyotypes of NP446Bmi-1/hTert cells were largely nearly diploid with the gain of chromosome 5 (Figure 3A). Interestingly, the NP550Bmi-1/hTert cells revealed a near tetraploid karyotype which was also confirmed by flow cytometric analysis of DNA content (Figure 3B). Nearly the whole set of chromosomes was duplicated in the immortalized NP550Bmi-1/hTert cells. In addition, there were a few other structural chromosomal changes observed, particularly involving chromosome 20. These structural changes may occur after tetraploidization as they were not detected in early passages. Detailed examination of DNA content by flow cytometry also revealed normal bimodal distribution of DNA content at G1/S and G2/M phase of primary NP550 cells (Figure 3B). At PD 27, an additional DNA peak could be detected at 8N DNA content revealing the emergence of a G2/M tetraploid population (8.64%) of NP550Bmi-1/hTert cells (Figure 3B). At PD 85, the entire population of NP550Bmi-1/hTert cells revealed a 4N DNA population (76.04%) at G1 phase and 8N DNA population (10.54%) at G2/M phase, with 0% of cells with 2N DNA content at G1 phase, indicating completely takeover of cell population by the tetraploid population of Bmi-1/hTert-immortalized NP550 cells. The underlying events leading to this switching in ploidy in NP550Bmi-1/hTert cells are not completely understood. One speculation is that the tetraploidization of NP550Bmi-1/hTert may confer growth advantage to NP550Bmi-1/hTert cells and facilitated their immortalization. 

### Alteration of gene expression in Bmi-1/hTert-immortalized cells

**Figure 4:**
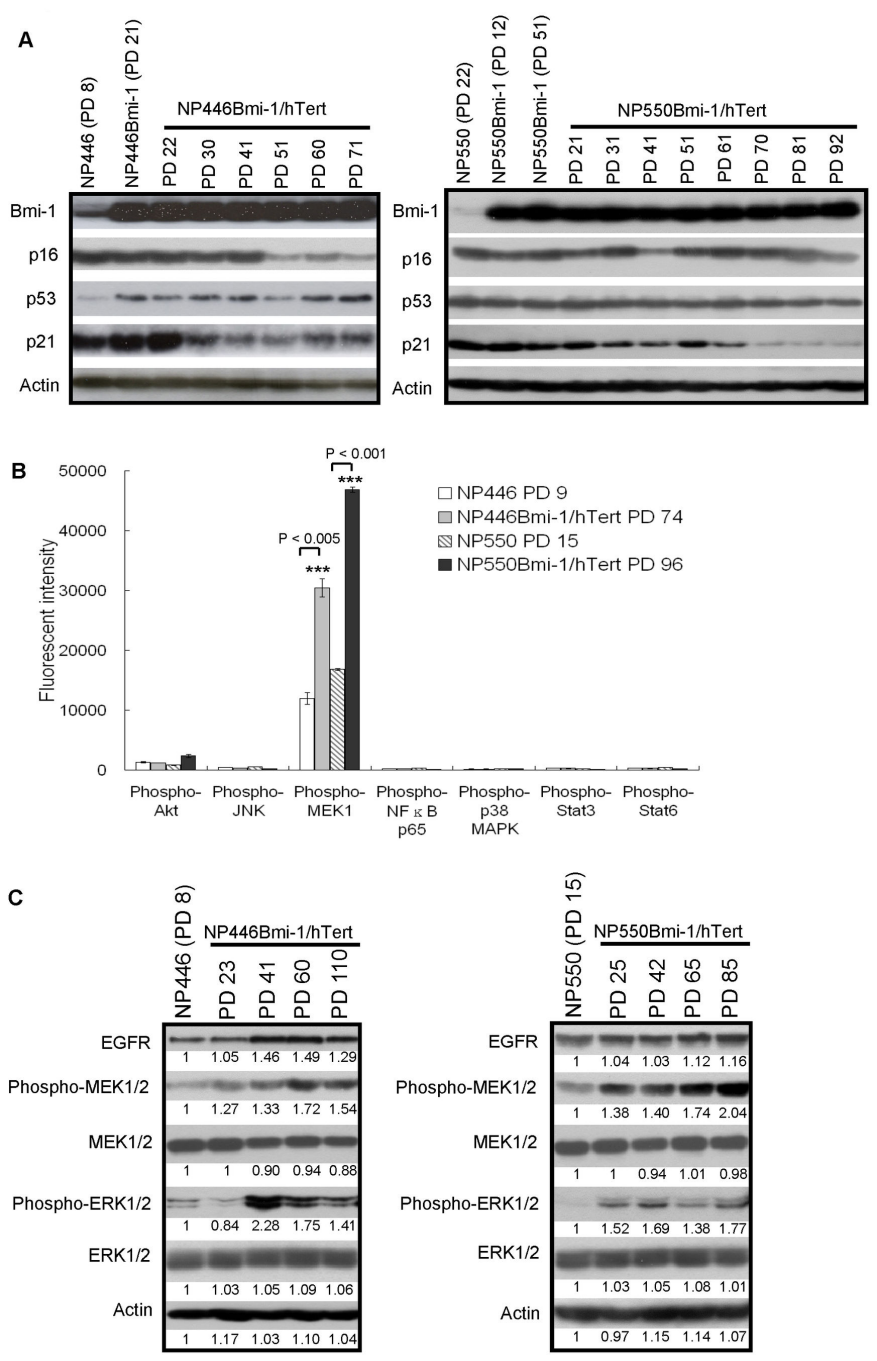
Alteration of gene expression in immortalized NPE cells. (A) Expression of genes involved in G1/S cell cycle entry. p16 and p21 were downregulated in the later passage of immortalized cells. (B-C) Activation of EGFR-MAPK signaling in immortalized cells. (B) Upregulation of phospho-MEK1 but not the other phosphorylated protein was detected in the immortalized cells compared to the control. Means and SDs were calculated from triplicate wells of two independent experiments. ***P < 0.005 and P < 0.001, Student t test. (C) The levels of EGFR, phospho-MEK1/2 and phospho-ERK1/2 expressions in cells were quantitated by ImageJ. Control expression in primary cells was set as 1. A gradual increase in EGFR and its downstream signaling, the phospho-MEK1/2 and phospho-ERK1/2, could be observed during immortalization of NP446Bmi-1/hTert and NP550Bmi-1/hTert.

We further examined the expression level of p16, p53 and p21 during the course of immortalization of NP446Bmi-1/hTert and NP550Bmi-1/hTert. Previous studies have reported that Bmi-1 could suppress p16 expression in immortalized cells. In both NP446 and NP550 cells, expression of *Bmi-1* alone or in combination with *hTert* did not reveal an immediate suppression of p16 expression (Figure 4A). We were only able to observe decrease of p16 expression at a later stage of immortalization. In NP446, decrease of p16 expression was only observed at PD 51 which was 30 passages after transduction of *Bmi-1*. The p16 levels fluctuated to some extents in NP550Bmi-1/hTert cells during the course of immortalization. Consistent downregulation of p16 was only observed at a much later passage (after PD 81). It could be concluded that suppression of p16 offers selective growth advantage to *Bmi-1*-expressing cells which facilitates their immortalization. The selective growth advantage of p16 silencing was also observed in our previous study using telomerase alone to immortalize primary NPE cells [3]. We did not observe obvious changes or trends of p53 expression during the course of immortalization in NP446 and NP550 cells (Figure 4A). In contrast, downregulation of p21 was observed during the course of immortalization of NP446 and NP550 cells.

### Upregulation of MAPK pathway in the Bmi-1/hTert-immortalized NPE cells

We then examined if specific signaling pathways are selected during immortalization of NPE cells by *Bmi-1* and *hTert* using the Bioplex phospho-protein detection assay. The signaling pathways examined included Akt, JNK, MAPK, NFκB, p38 MAPK, Stat3 and Stat6. The most prominent pathways activated in the immortalized NP446Bmi-1/hTert and NP550Bmi-1/hTert was MEK/MAPK signaling (Figure 4B). Activation of MAPK signaling in *Bmi-1/hTert*-immortalized NP446 and NP550 cells was further confirmed by Western blotting (Figure 4C). Notably, activation of MAPK signaling in NP446 and NP550 cells could be confirmed by Western blotting using specific phosphorylated antibodies for activated MEK1/2 and ERK1/2 at early passages, before decrease of p16 expression. We also observed a moderate increase in EGFR expression in both immortalized cells suggesting a selective advantage of MAPK activation for *Bmi-1/hTert*-immortalized cells. In NP446, activation of MAPK was not observed immediately after *Bmi-1* expression indicating that activation of MAPK signaling is not a direct action of *Bmi-1* or *hTert* and may represent a selected phenotype in *Bmi-1* and *hTert* immortalized cells. Our previous study has also shown the overexpression of *EGFR* and MAPK signaling during immortalization of NPE cells by *LMP1* and telomerase which is supportive of a role of MAPK activation in immortalization of primary NPE cells [12]. 

### Upregulation of Bmi-1 driven gene expression in immortalized NPE cells

**Figure 5:**
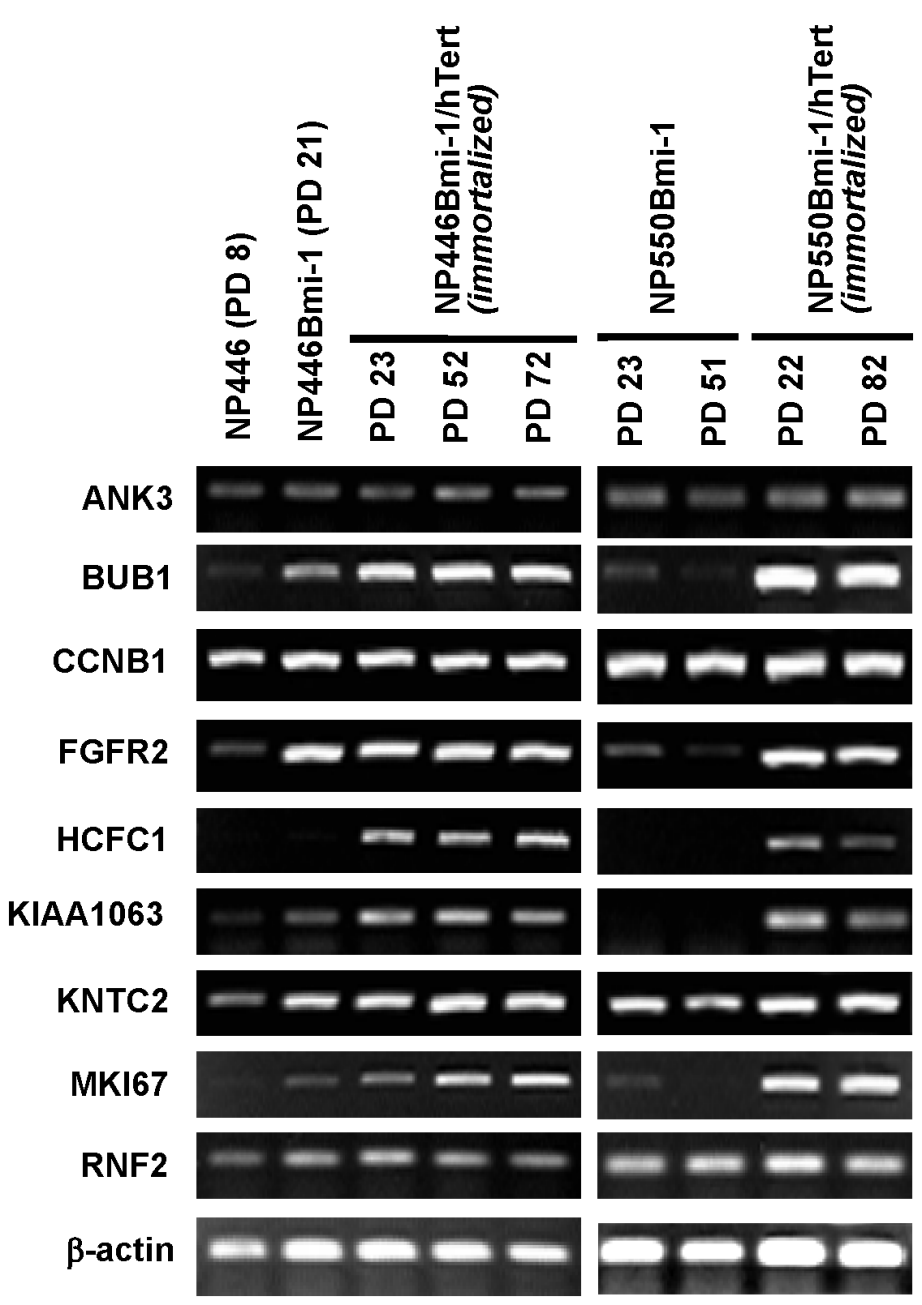
Expression of genes involved in Bmi-1 driven stemness pathway. All the stem cell-ness genes in the Bmi-1 driven pathway were upregulated in the immortalized cells and could be detected at both early and later passages of immortalization.

Bmi-1 has been shown to be involved in the maintenance of stem cells in tissues [20]. To confirm the functional activation of Bmi-1 after expression in the NPE cells, we have examined the expression status of the multiple genes downstream of *Bmi-1* activation. The expression of these genes have also been reported in *Bmi-1*-driven stem cell development pathway. The expression of these *Bmi-1*-driven genes at different passages of immortalized cells was examined by semi-quantitative RT-PCR analysis. In contrast to the cell cycle regulatory genes and MAPK signaling, we observed immediate activation of many of these Bmi-1 downstream gene targets in immortalized NP446Bmi-1/hTert and NP550Bmi-1/hTert cells (Figure 5). Expression of *Bmi-1* alone could also upregulate many of these genes in NPE cells before immortalization which may contribute to the extension of lifespan by *Bmi-1*. Interestingly, the expression levels of some genes appeared to be enhanced and stabilized after *hTert* expression.

### Essential role of p16 inactivation in the immortalization of NPE cells by Bmi-1 and hTert

**Figure 6:**
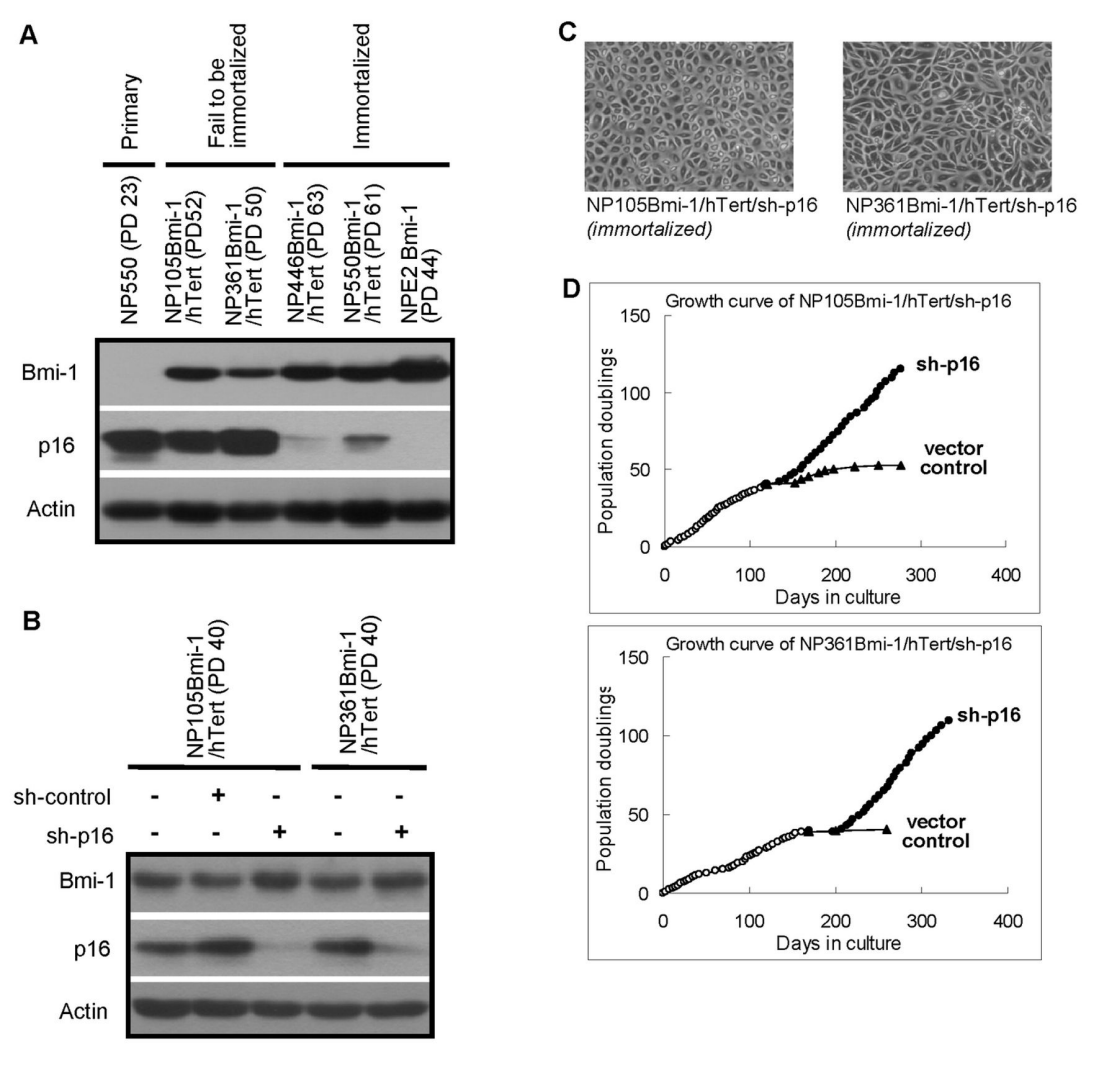
Immortalization of NP105 and NP361 cells by knocking down p16. (A) P16 expression in NP105 and NP361 cells. Primary cells (NP550) and cells that cannot be immortalized by Bmi-1/hTert (NP105 and NP361) expressed a high level of p16. (B-D) Knocking down p16 in NP105 and NP361 cells could lead to immortalization of both cell lines.

Our results showed that Bmi-1 could effectively extend the lifespan of primary NPE cells and inhibit onset of cellular senescence. In combination with telomerase activation, the ability to extend the lifespan of NPE cells was even more prominent. Nonetheless, expression of *Bmi-1* and *hTert* was only successful in immortalizing two out of four primary cultures of NPE cells. Additional events are required to achieve efficient immortalization of NPE cells. Examination of these NPE cells revealed that *p16* silencing may be a crucial event for immortalization of NPE cells by *Bmi-1* and *hTert*. In both NP446 and NP550 cells immortalized by *Bmi-1* and *hTert*, we observed suppression of *p16* at late passages. The downregulation of p16 may not be the direct action of *Bmi-1* as it was only observed at late passages. The role of *p16* in the onset of cellular senescence is well recognized. A consistently high level of p16 was observed in NP105 and NP361 cells which failed to be immortalized despite the effective expression of *Bmi-1* and activation of telomerase (Figure 6A). We proceeded to examine if knocking down p16 expression in NP105 and NP361 cells using a lentiviral plasmid expressing shRNA against *p16* might result in immortalization of these two NPE lines. Indeed, both NP105Bmi-1/hTert and NP361Bmi-1/hTert cells resumed their proliferation upon effective knock-down of p16 and achieved immortalization (Figures 6B, 6C and 6D). Hence we have effectively immortalized four out of four primary NPE cells using combination of *Bmi-1 overexpression, hTert* activation and *p16* silencing.

### EBV infection of the Bmi-1/hTert-immortalized cells

**Figure 7:**
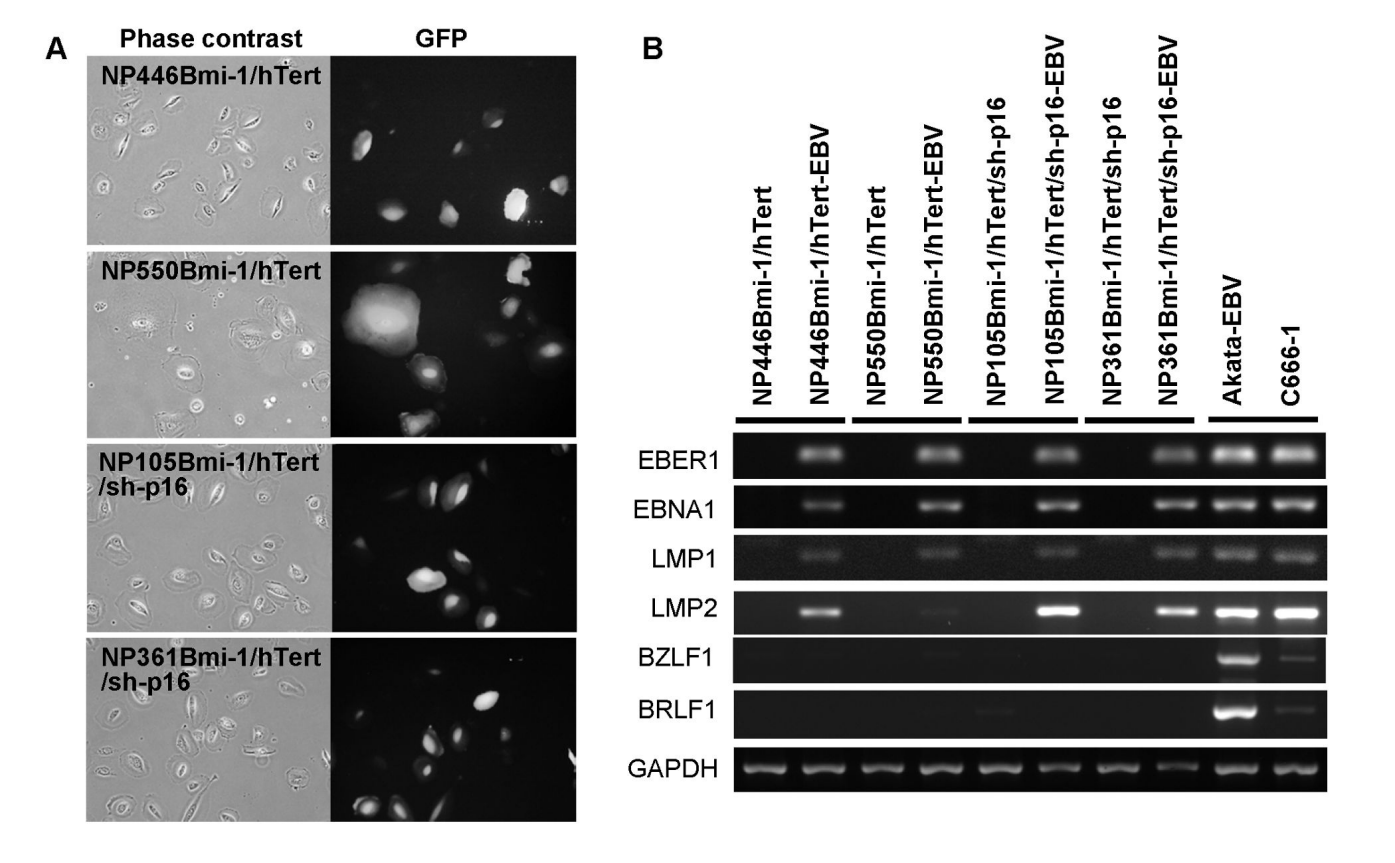
EBV infection of immortalized NPE cells. (A) EBV infection in the immortalized cells by fluorescent imaging of GFP expression. (B) The immortalized cells are susceptible to EBV infection. EBV-encoded genes could be detected in the immortalized cells after EBV infection.

EBV infection has long been postulated to play a critical role in NPC pathogenesis. The lack of representative cell systems for EBV infection has hindered the progress in the understanding of the role of EBV in NPC pathogenesis. A detail examination of the application of our immortalized NPE cells for EBV infection will be beyond the scope of this current study. It is essential to examine if the NPE cell lines immortalized by *Bmi-1, hTert* and silencing of p16 are susceptible for EBV infection. We have previously reported the successful EBV infection in a telomerase-immortalized NPE cell line [17]. Infection of immortalized NPE cells by EBV could be achieved by co-culturing with EBV-producing Akata cells. Alternatively, EBV infection could also be achieved using concentrated viral supernatant harvested from EBV-producing Akata cells. The EBV used to infect the NPE cells was GFP-tagged which facilitated the identification of EBV-infected cells by fluorescent imaging. Immortalized NPE cells were infected with EBV by both co-culture and direct cell free infection (Figure 7A and Table 1). Our results showed that the immortalized NPE cells could be effectively infected by either method. We were able to detect expression of multiple transcripts of EBV gene (EBER1, EBNA1, LMP1 and LMP2) in all the immortalized NPE cells established in this study (Figure 7B). The expression of EBV lytic genes (BZLF1 and BRLF1) was low or undetectable in our EBV-infected immortalized NPE cells. The profiles of EBV gene expression detected in infected NPE cells were representative of type II latent EBV infection which is characteristic of EBV infection in NPC. The dynamics of EBV gene expression in freshly infected and stably infected NPE cells are under current investigation.

**Table 1:** All the Bmi-1/hTert-immortalized NPE cells could be effectively infected by EBV.

	**EBV infection rate (% GFP positive)**	
	**Co-culture infection**	**Cell-free infection**
NP446Bmi-1/hTert	19.6	10.3
NP550Bmi-1/hTert	54.7	21.9
NP105Bmi-1/hTert/sh-p16	23.5	7.2
NP361Bmi-1/hTert/sh-p16	36.8	12.9

Both co-culture and direct cell free EBV infection could be achieved in the immortalized cells.

## Discussion

Overexpression of *Bmi-1* is common in many types of human cancer [5,9,21], including nasopharyngeal carcinoma [4]. Overexpression of *Bmi-1* has been reported in over one-third of NPC. *Bmi-1* immortalized NPE cells may provide representative cell models for study of NPC pathogenesis. The immortalization function of *Bmi-1* has been previously reported in multiple cell types. Earlier studies have reported that expression of *Bmi-1* alone could immortalize fibroblasts [22], human mammary epithelial cells [9] and human nasopharyngeal epithelial cells [4]. However, the immortalization action of *Bmi-1* appears to vary among cell lines from different origins [10]. Most importantly, expression of *Bmi-1* was often observed to be insufficient to immortalize primary human epithelial cells [10,23]. In this study, we also observed that expression of *Bmi-1* alone failed to immortalize primary human NPE cells and additional events are required. A recent study indicated that additional events such as inhibition of TGF-β signaling may also be involved in the extension of life span of primary epithelial cells after Bmi-1 expression [23].

In this study, the ability of *Bmi-1* to extend lifespan was confirmed in primary NPE cells supporting its role in NPC pathogenesis reported in our earlier study [4]. The underlying mechanisms involved in the extension of lifespan of primary epithelial cells by *Bmi-1* are not well characterized. Most studies attributed this property to the suppression action of p16 by *Bmi-1* [9,10]. However, in this study, effective suppression of p16 expression was not observed after expression of *Bmi-1*. Additional events may be involved in the extension of lifespan in *Bmi-1* expression cells.

To achieve immortalization, telomerase activation is essential. In earlier studies, the activation of telomerase was reported to be induced by Bmi-1 expression during immortalization of epithelial cells [8,9,11]. A low level of telomerase was observed in human mammary epithelial cells after expression of Bmi-1 but was not sufficient for immortalization. The ability of Bmi-1 to activate telomerase during immortalization may be cell-type dependent. We were not able to detect significant telomerase activation in all our primary NPE cells examined. Similarly, telomerase activation was also not observed in skin keratinocytes and small airway epithelial cells [10]. In oral keratinocytes immortalized by *HPV16 E6* and *Bmi-1*, telomerase activation was observed only after the cells have emerged from telomere erosion-induced crisis[11]. In our study, telomerase activation by *hTert* was required for efficient immortalization of primary NPE cells.

Expression of *Bmi-1* and *hTert* was only successful in the immortalization of two primary NPE cells (NP446 and NP550) out of four examined (50%). Characterization of NP446 and NP550 cells immortalized by Bmi-1 and hTert reviewed additional events may be involved during immortalization. One of the biological properties of *Bmi-1* is self-renewal and maintenance of human stem cells [5,24]. A comparative genomics approach has revealed genes driven by Bmi-1 which have been reported to be involved in maintenance of the stem cell properties [16]. In this study, we observed effective and immediate activation of *Bmi-1*-targeted genes after expression of *Bmi-1* in primary NPE cells, prior to suppression of *p16* expression. Their involvement in immortalization of NPE cells warrants further investigations. Furthermore, downregulation of the p21 protein and activation of MAPK signaling were observed in both *Bmi-1/hTert*-immortalized NP446 and NP550 cells suggesting their involvement in immortalization of NPE cells. Interestingly, we observed a change of ploidy of NP550 cells during immortalization by *Bmi-1* and *hTert*. The ploidy of primary NP550 cells, which was largely diploid, drifted to become near-tetraploid after immortalization by *Bmi-1* and *hTert*. The exact mechanism involved in the change of ploidy is under investigation and may involve activation of aurora kinase A (unpublished observation). Expression of *Bmi-1* appears to provide a selective growth advantage of these near-tetraploid NP550 cells. Aberrant mitosis leading to gross chromosomal rearrangement and ploidy changes may induce cell cycle arrest and apoptosis. *Bmi-1* expression has been shown to promote survival of cells to chemotherapeutic agents [25] and plays a role in DNA damage repair. Expression of Bmi-1 may promote survival of cells harboring aberrant chromosomal changes, which otherwise may trigger cellular apoptosis induced by mitotic abnormalities. Further investigations are warranted to determine if *Bmi-1* expression may promote survival of cells harboring genetic alterations. 

The examination of *p16* expression during the immortalization of primary NPE cells by *Bmi-1* reveals a complex pattern. The ability of Polycomb group proteins to suppress p16 expression for extension of *in vitro* lifespan of cells and eventually immortalization of fibroblasts has been previously reported [7,8,26]. Bmi-1 has been reported to suppress p16 expression through transcriptional repression [4,8]. The suppression of *p16* expression by *Bmi-1* may be cell type dependent. Suppression of *p16* expression was not prominent in NPE cells after expression of *Bmi-1* and was only observed at later passages after expression of *Bmi-1* and *hTert*. In NP446 cells, significant suppression of *p16* was only observed after PD 50 and may be a selective event to support *Bmi-1* and *hTert* immortalization of NPE cells. The *p16* expression levels in NP550 cells also fluctuated after *Bmi-1* and *hTert* expression. Consistent suppression of *p16* expression was only observed at late passages (after PD 81). In our previously reported study, *p16* deletion is a critical event selected by immortalized NPE cells after expression of telomerase [3]. Suppression of *p16* clearly offers a growth advantage for NPE cells and is actively selected during immortalization. In NP105 and NP361 cells, expression of *p16* remained high after ectopic expression of *Bmi-1* and *hTert*, and they failed to become immortalized. Silencing of *p16* expression was required to immortalize these two cell lines clearly indicating the importance of *p16* inactivation in immortalization of NPE cells. 

*Bmi-1* expression, telomerase activation and *p16* inactivation are all common events present in NPC [27]. The ability of Bmi-1 expression, telomerase activation and p16 silencing to efficiently immortalize primary NPE cultures has enabled us to establish immortalized NPE cells from high risk NPC population for NPC studies. Furthermore, NPE cells immortalized by combined action of *Bmi-1, hTert* and silencing of *p16* are susceptible to EBV infection and enable establishment of type II latent EBV infection characteristic of EBV infection in NPC. The role of EBV in NPC pathogenesis is still enigmatic at this stage. Events relating to infection of EBV and persistent of EBV infection in premalignant nasopharyngeal epithelium remain poorly defined. Efficient immortalization of primary NPE cells susceptible to EBV infection will facilitate investigations of EBV infection in NPC pathogenesis. 

## Supporting Information

Figure S1 **Activation of telomerase in primary nasopharyngeal epithelial cells.** Telomerase was activated in primary nasopharyngeal epithelial cells by transduction with hTert. However, immortalization could not be achieved.
(TIFF)

File S1 **Contains Tables S1 and S2.** Table S1: Information of primary nasopharyngeal epithelial cultures. Table S2: Highest population doublings that can be achieved by the primary nasopharyngeal epithelial cells expressing different genetic elements (Bmi-1, LMP1, cyclin D1 and CDK4). Cells expressing Bmi-1 could achieve the highest population doublings compared to those expressing other genetic elements.
(DOC)
