# Supplementary figures and images for: Efficient Immortalization of Primary Nasopharyngeal Epithelial Cells for EBV Infection Study
Source: PLoS One. 2013 Oct 22;8(10):e78395. doi: 10.1371/journal.pone.0078395 (PMC3805559; doi:10.1371/journal.pone.0078395)

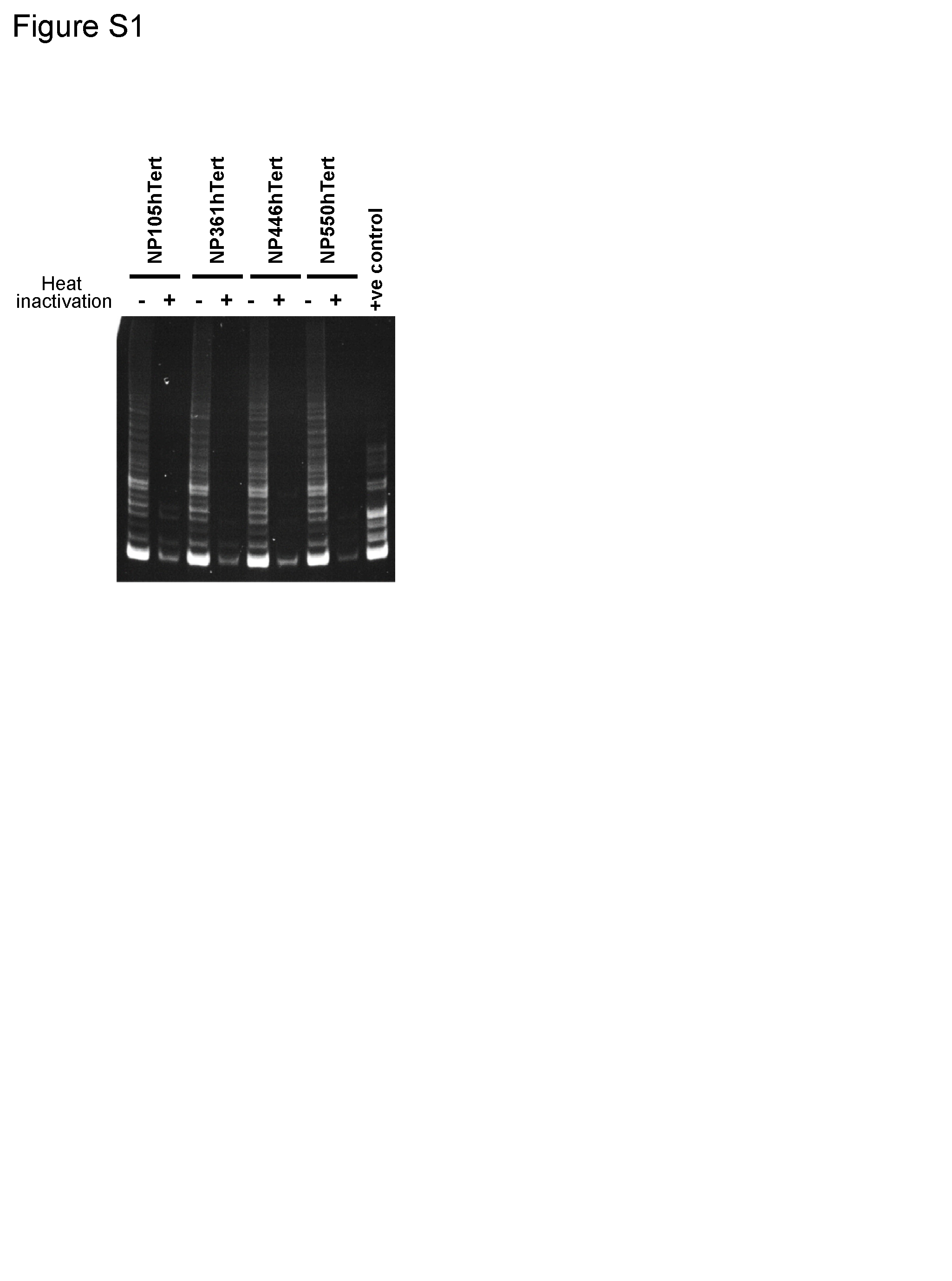

Supplement: Figure S1 — Activation of telomerase in primary nasopharyngeal epithelial cells. Telomerase was activated in primary nasopharyngeal epithelial cells by transduction with hTert. However, immortalization could not be achieved. (TIFF) [file pone.0078395.s001.tiff]
