# Supplementary material for: Efficient Immortalization of Primary Nasopharyngeal Epithelial Cells for EBV Infection Study
Source: PLoS One. 2013 Oct 22;8(10):e78395. doi: 10.1371/journal.pone.0078395 (PMC3805559; doi:10.1371/journal.pone.0078395)
Supplement: File S1 — Contains Tables S1 and S2. Table S1: Information of primary nasopharyngeal epithelial cultures. Table S2: Highest population doublings that can be achieved by the primary nasopharyngeal epithelial cells expressing different genetic elements (Bmi-1, LMP1, cyclin D1 and CDK4). Cells expressing Bmi-1 could achieve the highest population doublings compared to those expressing other genetic elements. (DOC) [file pone.0078395.s002.doc]

**Supporting Information**

# Table S1. Information of primary nasopharyngeal epithelial cultures.

| Primary nasopharyngeal epithelial cultures | Origin of nasopharyngeal biopsies | Histopathological properties | Passage number at senescence |
| --- | --- | --- | --- |
| NP361 | Nasopharyngeal biopsy from NPC patient | Tumor-free | 20 |
| NP550 | Nasopharyngeal biopsy from NPC patient | Tumor-free | 24 |
| NP105 | Nasopharyngeal biopsy from patient underwent tonsillectomy | Tumor-free | 6 |
| NP446 | Nasopharyngeal biopsy from patient underwent tonsillectomy | Tumor-free | 10 |

# Table S2. Highest population doublings that can be achieved by the primary nasopharyngeal epithelial cells expressing different genetic elements (Bmi-1, LMP1, cyclin D1 and CDK4).

|  | | Highest population doublings that can be achieved by the cells |
| --- | --- | --- |
| **NP361** | Bmi-1 | 29 |
|  | LMP1 | 24 |
|  | Cyclin D1 | 20 |
|  | CDK4^R24C^ | 21 |
| **NP446** | Bmi-1 | 27 |
|  | LMP1 | 16 |
|  | Cyclin D1 | 15 |
|  | CDK4^R24C^ | 11 |
| **NP550** | Bmi-1 | 57 |
|  | LMP1 | 15 |
|  | Cyclin D1 | 18 |
|  | CDK4^R24C^ | 15 |

Cells expressing Bmi-1 could achieve the highest population doublings compared to those expressing other genetic elements.
